# Immunomodulatory role of phagocyte-derived chloramines involving lymphocyte glutathione

**DOI:** 10.1155/S0962935193000328

**Published:** 1993

**Authors:** Véronique Witko-Sarsat, Anh Thu Nguyen, Béatrice Descamps-Latscha

**Affiliations:** INSERM U25 Hôpital Necker 161 rue de Sèvres Paris 75743 France

## Abstract

This study shows that human lymphocytes markedly decrease chloramines (long-lived oxidants) generated by polymorphonuclear neutrophils (PMN) after stimulation by phorbol-myristate-acetate or opsonized zymosan. In a cell-free model, reduced glutathione (GSH) scavenged chloramines, giving rise to oxidized glutathione (GSSG). In the cell system, treatment of lymphocytes with autologous PMN-derived chloramines induced a profound decrease in their total and reduced glutathione (GSH) content and markedly inhibited their proliferate responses to concanavalin-A and, to a lesser extent, phytohaemagglutinin. It is concluded that (i) lymphocytes may play a defensive role against phagocyte-derived oxidative stress by scavenging chloramines, and (ii) as this effect which is mediated by GSH affects lymphocyte proliferative responses, it may help to elucidate the still obscure mechanisms of oxidative stress associated immunodeficiency.


Mediators of Inflammation 2, 235–241 (1993)

### Immunomodulatory role of phagocyte-derived chloramines involving lymphocyte glutathione

Véronique Witko-Sarsat, Anh Thu Nguyen and Béatrice Descamps-Latscha[CA]

INSERM U25, Hôpital Necker, 161 rue de Sèvres, 75743 Paris, France

[CA] Corresponding Author

**THIS study shows that human lymphocytes markedly decrease chloramines (long-lived oxidants) generated by polymorphonuclear neutrophils (PMN) after stimulation by phorbol-myristate-acetate or opsonized zymosan. In a cell-free model, reduced glutathione (GSH) scavenged chloramines, giving rise to oxidized glutathione (GSSG). In the cell system, treatment of lymphocytes with autologous PMN-derived chloramines induced a profound decrease in their total and reduced glutathione (GSH) content and markedly inhibited their proliferate responses to concanavalin-A and, to a lesser extent, phytohaemagglutinin. It is concluded that (i) lymphocytes may play a defensive role against phagocyte-derived oxidative stress by scavenging chloramines, and (ii) as this effect which is mediated by GSH affects lymphocyte proliferative responses, it may help to elucidate the still obscure mechanisms of oxidative stress associated immunodeficiency.**

**Key words:** Chloramine, Concanavalin-A, Glutathione, Human phagocyte, Immunodeficiency, Lymphocyte proliferation, Phytohaemagglutinin, Polymorphonuclear neutrophils, Taurine–chloramine

### Introduction

Polymorphonuclear neutrophils (PMN) and monocytes activated by phagocytic or soluble stimuli produce large amounts of microbicidal oxidants that play a key role in host defences against pathogens.[1–2] This metabolic response, known as the 'respiratory burst', involves two enzyme pathways: (i) NADPH oxidase activation leads to the reduction of molecular oxygen to superoxide anion and the subsequent generation of hydrogen peroxide,[3] and (ii) the activity of neutrophil myeloperoxidase (MPO), an enzyme located in the azurophilic granules that are released following degranulation, catalyses the reaction of $H_2O_2$ with chloride, leading to the formation of other oxidants such as hypochlorous acid and chloramines.[4] These latter, known as long-lived oxidants,[5] may be mediators of phagocyte-induced oxidative damage[6] as they are either released or generated via the extracellular MPO in the extracellular environment. Their ability to inactivate protease inhibitors such as $\alpha_1$-antiproteinase[7] is one way in which chloramines could potentiate deleterious effects in inflammatory processes. The presence of chloramines in biological fluids seems to be highly dependent on the status of antioxidant defences.[8] Natural killer activity, lymphocyte proliferation in response to mitogens, and immunoglobulin and interleukin-2 secretion are apparently affected by $H_2O_2$ and MPO.[9,10]

The aim of this study was to investigate potential interactions between PMN-derived chloramines and lymphocytes. Glutathione,[11] the major non-protein thiol, is present in virtually all cell types and is involved in numerous biological functions, acting as a co-enzyme, antioxidant, and regulatory molecule in cell cycle initiation and progression[12] and microtubule formation.[13] In addition, glutathione content is important in lymphocyte activation and proliferation.[14,15]

The authors therefore investigated (1) the influence of lymphocytes on chloramine release by PMN, (2) the biochemical mechanism of this action, and (3) the biological consequences of such an interaction on lymphocyte functions.

### Materials and Methods

*Chemicals:* Sulphosalicylic acid and vinyl pyridine were from Aldrich (Strasbourg, France), glutathione (reduced (GSH) and oxidized (GSSH)), glutathione reductase and NADPH from Boehringer (Meylan, France), red phenol-free Hanks' balanced salt solution (HBSS) from Eurobio (Paris, France), RPMI 1640 medium from GIBCO, phytohaemaglutinin A (PHA) from Wellcome S.A. Division Diagnostic (Paris, France), concanavalin A (Con A) from Miles Scientific (Naperville, IL), Ficoll–Hypaque from Pharmacia LKB Biotechnology Inc. (Piscataway, NJ), acetic acid from Prolabo (Paris, France), plasmagel from Roger Bellon Laboratoire (Paris, France), and chloramine-T (N-chloro-*p*-toluene-sulphonamide sodium salt), DTNB (5,5′-dithio-bis-2-nitrobenzoic acid), phorbol-myristate-acetate (PMA, 4$\beta$-phorbol,12$\beta$-myri-

© 1993 Rapid Communications of Oxford Ltd state 13α-acetate), potassium iodide, taurine, triethanolamine, Trypan blue and zymosan A (*Saccharomyces cerevisiae*) were from Sigma Chemical Co. (St Louis, MO).

*Cell preparations:* Normal PMN were isolated from heparinized (Liquemine Roche, 10 U/ml) venous blood of healthy donors (Centre de Transfusion de l'Hôpital Necker).[16] Briefly, PMN were separated from erythrocytes by plasmagel sedimentation, followed by Ficoll–Hypaque density centrifugation. Residual erythrocytes were lysed by treating the cell pellet with a lysis buffer containing ammonium chloride. The purified PMN were then resuspended in HBSS at $10^6$ cells/ml. Mononuclear cells obtained after Ficoll–Hypaque density centrifugation were added to Falcon 3047 tissue culture dishes (Falcon Labware, Becton Dickinson and Co., Oxnard, CA) and incubated for 60 min at 37°C in humidified 95% air/5% $CO_2$ for monocytes to attach. Lymphocytes (non-adherent cells) were then harvested, washed and seeded at the required density.

*Chloramine production and PMN supernatant preparation:* PMN incubation and taurine–chloramine production were carried out as described previously.[17] Briefly, PMN were incubated for 1 h at 37°C with constant gentle shaking in the absence (controls) or presence of PMA, (final concentration 1 μg/ml), or opsonized zymosan (OZ) particles (2 mg/ml) prepared as described elsewhere.[16] PMN incubation was performed in the absence or presence of autologous lymphocytes. Taurine–chloramine formation was enhanced by adding taurine (15 mM) to the incubation medium. At the end of the incubation period the samples were centrifuged at 4°C (1 200 × *g* for 10 min) the supernatants stored at −20°C for testing on the following day. OZ-stimulated PMN supernatants were used as a source of biological chloramines. To avoid cross-reactions with degranulation proteins, supernatants were filtered with 3-kDa cut-off Centricon filters (Amicon, Grace, France). Filtration did not affect the chloramine concentration (data not shown).

*Colorimetric chloramine determination in PMN supernatants:* Chloramines were determined by colorimetric measurement of the triiodide ion formed by the oxidation of potassium iodide (KI)[17] in 96-well microtitre plates (Falcon Labware, Becton Dickinson and Co.; Oxnard, CA). Two hundred μl of sample (supernatant or the chloramine-T standard solution) was placed in each well. Ten μl of 1.16 M KI was then added, followed by 20 μl of acetic acid 2 min later. The absorbance of the reaction mixture was immediately read at 340 nm in a microplate reader (Model MR 5000, Dynatech, France) against a blank containing 200 μl HBSS, 10 μl KI and 20 μl acetic acid. Absorbance at 340 nm follows Beer's law within the range of 0–100 μM, assuming an extinction coefficient of 26 $mmol/cm^1$.

*Lymphocyte treatment with chloramines:* Lymphocytes ($10^6$ cells/ml in HBSS) were exposed either to autologous PMN-derived chloramines (determined by the colorimetric assay) or to synthetic chloramine (chloramine-T) in HBSS (without serum in order to avoid chloramine scavenging or interference in the glutathione assay). After 1 h of incubation at 37°C under gentle shaking, lymphocytes were centrifuged and the chloramine concentration was assayed in the supernatant; the cell pellet was resuspended in 5% serum RPMI 1640 medium for cell culture experiments or in HBSS for GSH determination.

*Cell viability:* After exposure to chloramines, lymphocyte viability was measured in terms of Trypan blue exclusion. Twenty μl of Trypan blue (0.5%) was added to 80 μl of lymphocyte suspension ($10^6$ cells/ml).

*Glutathione determination:* The total GSH concentration in lymphocytes was determined by means of an enzymic recycling assay.[18] After pretreatment with chloramines, lymphocytes ($10^6$/tube) were centrifuged and resuspended in 200 μl of 5% sulphosalicylic acid and sonicated. After centrifugation (10 min, 20 000 × *g*), GSH was measured in the supernatant. The assay was performed in microtitre plates as follows: in each well, 140 μl of NADPH (0.3 mM), 30 μl of DTNB (5 mM) and the sample (or GSH standard solution) were mixed and incubated for 10 min at 30°C. The optical density at 405 nm was measured 1 min after triggering the reaction with 20 μl of glutathione reductase (2 U/ml). The oxidized GSH concentration (GSSG) was determined after sample derivatization by adding 2 μl of vinyl pyridine and 8 μl of triethanolamine to 100 μl of sample (or GSSG standard solution). GSSG was then determined in the same manner as GSH. The concentration of reduced GSH was calculated as the difference between total GSH and GSSG.

*Mitogen-induced lymphocyte proliferation:* Mitogen assays were carried out in flat-bottomed, 96-well microtitre plates using RPMI 1640 medium containing 5% heat-inactivated foetal calf serum (Flow Laboratories, Irvine, Scotland), 2 mM L-glutamine, 100 U/ml penicillin and 100 μg/ml streptomycin. Each well contained 2 × $10^5$ lymphocytes and the proliferative response was induced by adding either Con-A (1 μg/ml) or PHA (0.05 μg/ml) in a final volume of 200 μl. Plates were incubated for 44 h at 37°C in a humidified atmosphere containing 5% $CO_2$. Cultures were pulsed with 5 μCi (1 Ci = 37 GBq) of [$^3$H]-thymidine (5 Ci/mmol,

CEA, Saclay, France), harvested 4 h later and counted for beta emission. Each value of proliferation has been determined in triplicate.

*Statistical analysis:* Results (mean ± S.E.M.) were compared using Student's two-tailed *t*-test (paired or unpaired, as appropriate). Differences were considered significant when the $p$ value was below 0.05. Correlation coefficients were calculated by using simple regression analysis.

### Results

*Influence of lymphocytes on PMN chloramine release:* Chloramine secretion by a purified PMN suspension ($10^6$ cells/ml) in the absence and presence of autologous lymphocytes ($10^6$/ml) was compared first. As shown in Fig. 1, co-incubation of PMN with viable lymphocytes significantly decreased the chloramine concentration in supernatants of PMA- and OZ-stimulated PMN. This effect was even more pronounced when lymphocytes were lysed prior to incubation. In the absence of PMN, lymphocytes exerted a significant scavenging effect on both PMN-derived chloramines and chloramine-T (Fig. 2). In contrast, the supernatant of lymphocytes had no effect on PMN-derived chloramines (data not shown).

*Role of glutathione in lymphocyte scavenging of chloramines:* To investigate the molecular basis of chloramine scavenging by lymphocytes, the reactivity of glutathione with chloramines in cell-free conditions was tested. Figure 3 shows that GSH but not GSSG

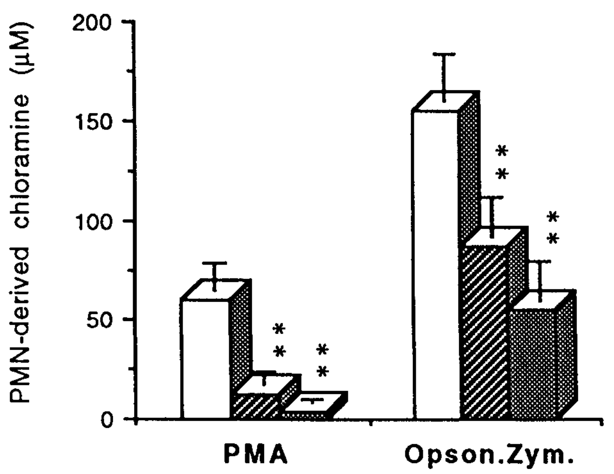

FIG. 1. Effect of co-incubation of lymphocytes with PMN stimulated to generate taurine–chloramine. PMN ($10^6$/ml) were stimulated by either PMA (1 µg/ml) or opsonized zymosan (2 mg/ml) to produce taurine–chloramine. In the absence of a stimulus, no chloramines are detectable in the supernatant (data not shown). Either viable or lysed lymphocytes ($10^6$/ml) were added to the PMN at the beginning of the incubation. Data are the mean ± S.E.M. of five experiments with different donors. ** $p < 0.01$ (difference with PMN only). □, PMN only; ▨, +viable lymphocytes; ▩, +lysed lymphocytes.

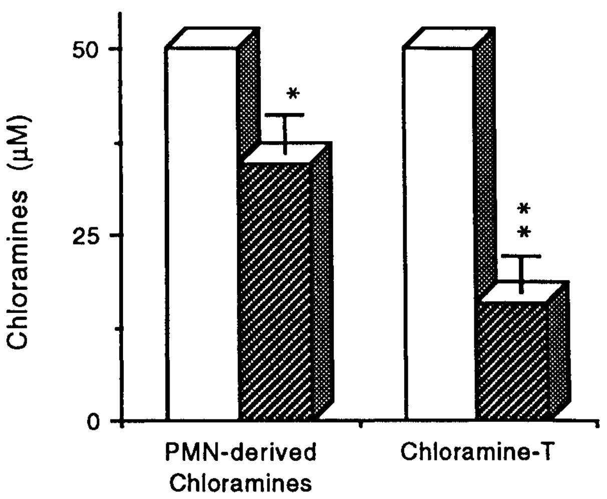

FIG. 2. Effect of lymphocytes on PMN-derived chloramines and on a synthetic chloramine (chloramine-T). PMN-derived chloramines were obtained after stimulation of PMN ($10^6$/ml) with opsonized zymosan (2 mg/ml) in the presence of taurine as described in Materials and Methods. The supernatants containing taurine–chloramine were filtered on Centricon 3 kDa. Chloramine concentration was adjusted to 50 µM for the experiment. Chloramine-T was dissolved in HBSS. Data are the mean ± S.E.M. of six experiments with different donors. * $p < 0.05$, ** $p < 0.01$ (difference with no lymphocytes). □, no lymphocytes; ▨, + lymphocytes.

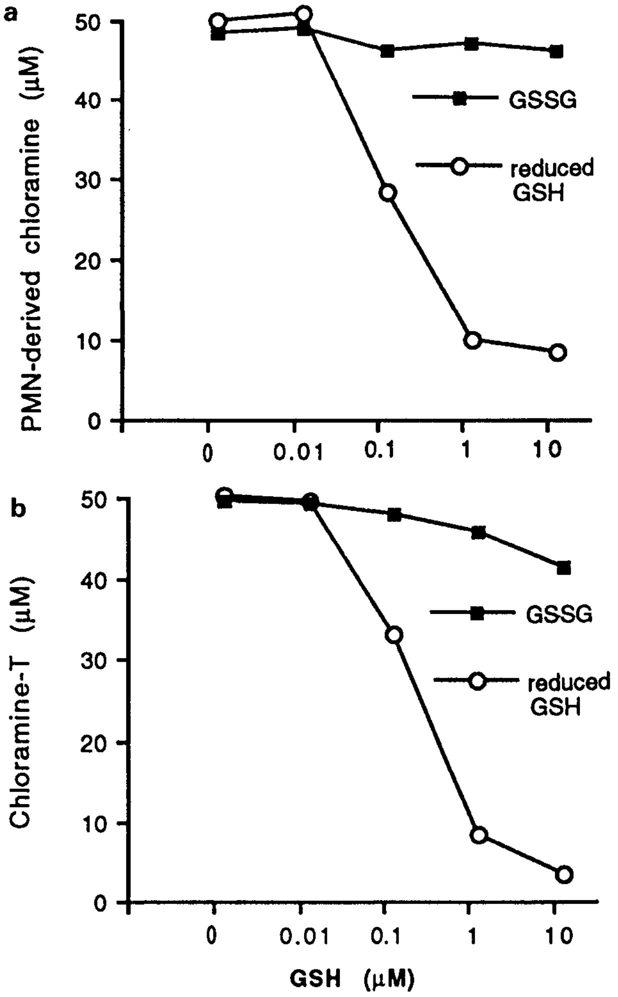

FIG. 3. Scavenging effect of GSH and GSSG on PMN-derived chloramines (a) and chloramine-T (b). Chloramine concentrations were assessed after 1 h incubation with GSH or GSSG at desired concentrations in HBSS. Each chloramine concentration has been determined in triplicate.

had a concentration dependent inhibitory effect on both chloramine-T and PMN derived chloramines. In order to investigate whether the chloramine scavenging effect of lymphocytes involved intracellular GSH, lymphocyte glutathione content before and after 1-h exposure to either chloramine-T or PMN derived chloramines were compared. As shown in Fig. 4, chloramines significantly decreased the lymphocyte glutathione content in a concentration dependent manner. Although this decrease was observed with both total and reduced GSH in the case of PMN derived chloramines (Fig. 4a), it was highly significant only in the case of chloramine-T (Fig. 4b).

*Effect of chloramines on mitogen-induced lymphocyte proliferation:* To obtain further evidence of an interaction between chloramines and lymphocyte functions, lymphocyte proliferative responses to mitogens were studied following exposure to chloramines. In this part of the study, only OZ was used to stimulate PMN chloramine production since PMA may have a direct effect on lymphocyte proliferation. In addition, supernatants of OZ-stimulated PMN were filtered to remove proteins with molecular weights greater than 3 kDa. Table 1 shows that chloramines did not affect lymphocyte viability, at least up to 50 $\mu$M. In the absence of mitogen, neither synthetic nor PMN derived chloramines significantly affected lymphocyte [$^3$H] thymidine incorporation. Fig. 5a shows the influence of PMN derived chloramines on lymphocyte proliferation induced by Con-A and PHA.

**Table 1.** Effect of chloramines on lymphocyte viability measured by Trypan blue exclusion

| | | Chloramine concentration ($\mu$M) | | | | |
|---|---|---|---|---|---|---|
| | | PMN-derived | | Chloramine-T | | |
| | Control 0 | 10 | 50 | 10 | 50 | 100 |
| Mortality % ($n = 3$) | 4.9 ±1.97 | 4.1 ±0.33 | 6.4 ±1.48 | 10.3 ±4.36 | 7.6 ±2.64 | 16.08 ±5.95 |

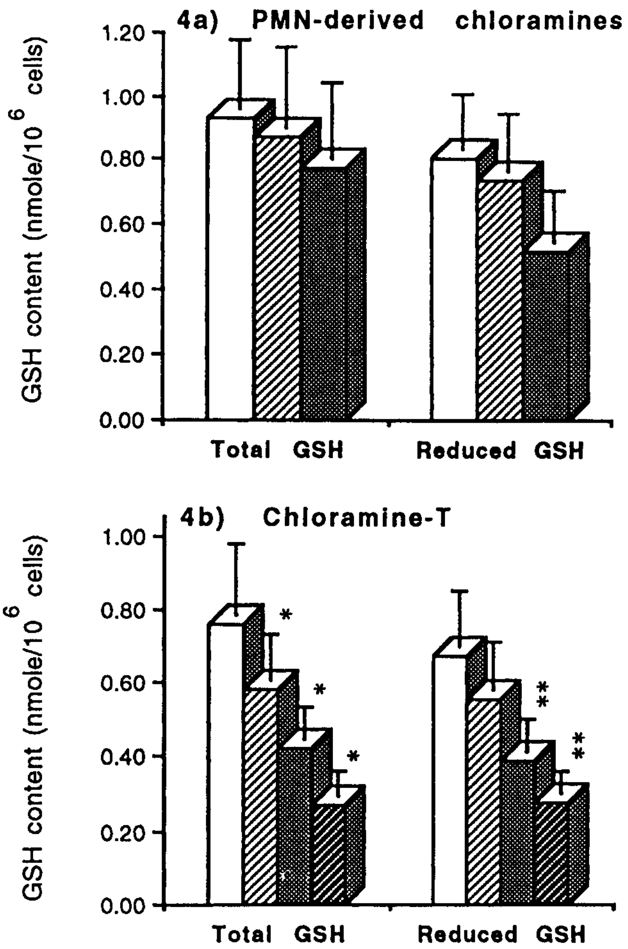

FIG. 4. Effect of PMN-derived chloramines (4A) or chloramine-T (4B) on lymphocyte GSH content. Total or reduced GSH content of $10^6$ lymphocytes was measured after 1 h exposure to chloramine. Data are the mean ± S.E.M. of six experiments with different donors for PMN-derived chloramines and of eight experiments for chloramine T. *$p < 0.05$, **$p < 0.01$ (difference with controls). □, Chlor 0 $\mu$M; ▨, Chlor 10 $\mu$M; ■, Chlor 50 $\mu$M; ■, Chlor 100 $\mu$M.

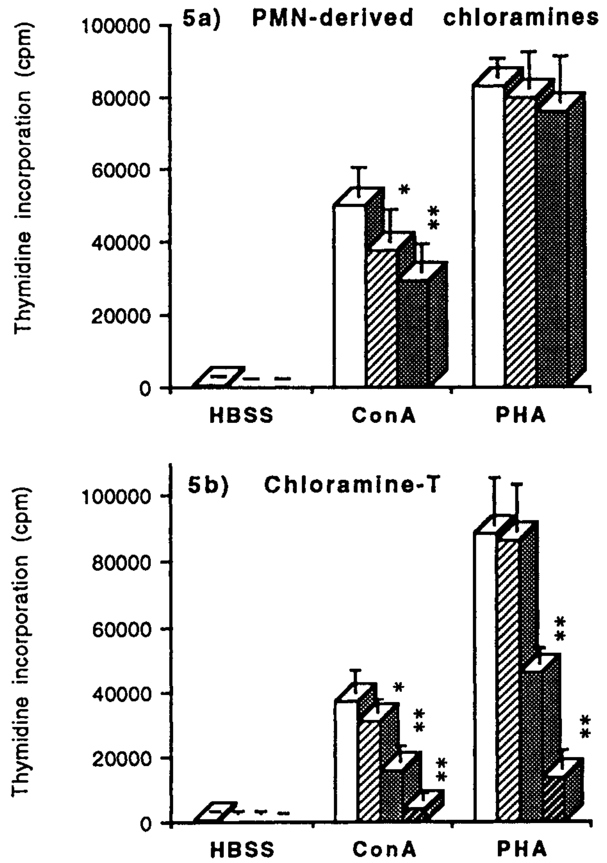

FIG. 5. Effect of PMN-derived chloramines (5A) or chloramine-T (5B) on mitogen-induced lymphocyte proliferation. After 1 h exposure to chloramines in HBSS, lymphocytes were cultured in RPMI medium ($2 \times 10^5$ cells/well) and proliferative responses were induced by adding either Con-A (1 $\mu$g/ml) or PHA (0.05 $\mu$g/ml). Proliferation response was evaluated by pulsing the culture with [$^3$H]thymidine. The y axis indicates the uptake of [$^3$H]thymidine by the cells in cpm during the last 4 h of 2-day cultures. Data are the mean ± S.E.M. of eight experiments with different donors for PMN-derived chloramines and of five experiments for chloramine-T. *$p < 0.05$, **$p < 0.01$ (difference with no chloramines). □, Chlor 0 $\mu$M; ▨, Chlor 10 $\mu$M; ■, Chlor 50 $\mu$M; ■, Chlor 100 $\mu$M.

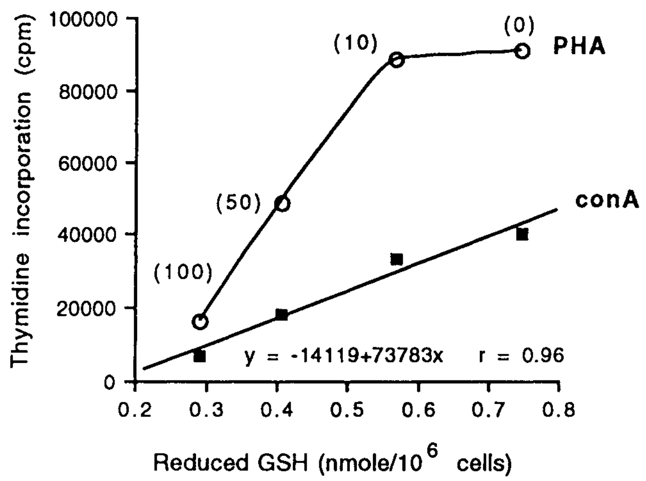

FIG. 6. Correlation between lymphocyte reduced GSH content and the mitogen-induced proliferation after lymphocyte exposure to chloramine-T (concentration given in parentheses). Each value of reduced GSH content and of mitogen-induced proliferation (Con-A or PHA) is the mean of five different experiments performed on different donors. ○, PHA; ■, Con-A.

Significant inhibition was observed only when lymphocyte proliferation was induced by Con-A (respectively 28 and 44% for chloramine concentrations of 10 and 50 $\mu$M). On the other hand, chloramine-T (Fig. 5b) induced a significant concentration dependent inhibition regardless of the mitogen used, for chloramine-T concentration greater than 10 $\mu$M. This is confirmed by the correlation ($r = 0.96$) found between lymphocyte GSH content and proliferative responses in case of stimulation by Con-A (Fig. 6). In contrast, PHA-induced proliferative response at low chloramine concentration (10 $\mu$M) seems to be less sensitive to chloramine and thus less dependent on glutathione availability.

### Discussion

This study shows that chloramines, described as long-lived oxidants, may be important immunomodulatory agents, the effect of which is mediated by the ubiquitous thiol-containing molecule, glutathione.[11]

The results suggest a potentially important scavenging role of lymphocytes for PMN derived chloramines, which are thought to be exported into the surrounding environment, and to potentiate PMN-induced oxidative damage.[19] The physiological relevance of this scavenging mechanism needs to be determined while the possible involvement of other plasma antioxidants[20] and other cell types, like red blood cells, which contain glutathione and which could compete for chloramines might also be considered. However, in inflammation-induced oxidative stress, when plasma antioxidant defence may be overwhelmed, lymphocytes might play an important role in oxidant scavenging. Both viable and lysed lymphocytes scavenged chloramines, indicating that the effect does not require protein synthesis or other metabolic pathways but rather involves preformed molecules that are more accessible after cell disruption.

Other studies have demonstrated the critical role of glutathione on oxidant-mediated cell lysis. Inhibition of the GSH redox cycle enhances tumour cell lysis induced by both PMA activated macrophages and $H_2O_2$ generated in the glucose–glucose oxidase (GGO) system. In that study, the content of glutathione correlated with the susceptibility of six murine tumour cell lines to lysis by a flux of $H_2O_2$;[21] moreover, such inhibition increases GGO mediated lysis of endothelial cells.[22] In addition, increased sensitivity of pulmonary vascular endothelial cells to $H_2O_2$ after tumour necrosis factor-$\alpha$ (TNF-$\alpha$) exposure, is mediated by a decrease in cellular glutathione.[23] In a cell-free system, it was found that only the reduced form of glutathione is able to scavenge both chloramine-T and PMN derived chloramines. Interestingly, the reaction of chloramines with GSH has recently been studied in a model of liver injury.[24] The authors found that chloramines (monochloramine and taurine–chloramine) were efficiently detoxified in the liver by intracellular GSH, inducing GSH depletion and a subsequent increase in perfusion pressure and a decrease in bile flow. As a result, they suggested that chloramines could contribute to PMN-induced organ injury through a GSH mediated pathway.

Our results indicate that both the total and reduced glutathione content of lymphocytes were reduced in a concentration dependent manner after chloramine challenge. Reduced glutathione is able to scavenge chloramines, giving rise to GSSG; GSSG would subsequently be reduced by intracellular glutathione reductase and reconverted to GSH. Under conditions of continuous exposure to chloramines, GSSG may accumulate because the rate of GSSG formation exceeds that of its reduction;[11] GSSG can then form mixed disulphides with intracellular or extracellular proteins, resulting in a net loss of total glutathione, as seen in this study. The lymphocyte total glutathione content observed (mean $\pm$ S.E.M., $0.7 \pm 0.15$ nmol/$10^6$ cells) is in the range of concentrations reported in T cells by other groups using the same GSH assay.[12–14]

Given the multiple roles of glutathione in cell functions, especially in lymphocytes, the consequences of direct chloramine exposure for lymphocyte proliferation were investigated. Lymphocyte proliferation was efficiently inhibited by chloramine-T, regardless of the mitogen used. In the case of PMN derived chloramines, only Con-A-stimulated lymphocytes showed significantly inhibited proliferation. These results suggest that the lymphocyte proliferative response to Con-A is more dependent on the glutathione concentration than that induced by PHA. In addition, chloramine-T was much more efficient than PMN derived chloramines in decreasing both the reduced and oxidized glutathione content of lymphocytes. In lymphocyte proliferation experiments, the same difference was observed. Although PMN supernatants were filtered to remove proteins with a molecular weight greater than 3 kDa, low molecular weight antioxidants could interfere with the effect of biological chloramines, this interference being overcome at high chloramine concentrations. Lymphocyte functions show different degrees of susceptibility to oxidative injury by MPO and $H_2O_2$: PHA- and Con-A-induced proliferation displayed intermediate degrees of susceptibility between pokeweed induced proliferation and antibody formation, this latter function being the most sensitive to oxidative injury.[9] These latter authors used a GGO as the $H_2O_2$-generating system in the presence or absence of MPO. At a rate of production of 60 μmol/ml/min of $H_2O_2$, the lymphocytes were 90% viable. This is compatible with our results showing no effect of chloramines on lymphocyte viability when the chloramine concentration was less than 50 μM and there was only a small loss of viability when the concentration reached 100 μM. The experimental conditions in the two studies were very similar.

Although the precise role of GSH in lymphocyte activation remains unclear, GSH synthesis is clearly essential to maintain normal lymphocyte proliferation and cytokine metabolism. As direct relationships exist between the proliferative response and glutathione availability,[15] lymphocyte proliferation can be enhanced by providing excess glutathione, cysteine or 2-mercaptoethanol, while it is strongly inhibited by L-buthionine-(*S*,R)-sulphoximine (BSO), a specific and irreversible inhibitor of γ-glutamyl cysteine synthetase that limits the quantity of intracellular GSH available during the activation process. This has been demonstrated both with T cells and purified large granular lymphocytes.[25] GSH enhances the effect of IL-2 and IL-4 on replication and [$^3$H]thymidine incorporation of IL-2 and IL-4 dependent cells, respectively, *in vitro*. This potentiating effect of GSH is accompanied by an increase in the intracellular GSH level and an enhancement of binding, internalization and degradation of both cytokines.[26,27]

This study provides some insights into the molecular mechanisms involved in the effect of oxidants on mitogen-induced lymphocyte proliferation and identifies a possible mechanism for the immunodepression observed following oxidative stress. Indeed, there was a close correlation between lymphocyte proliferation and GSH content after chloramine-T exposure, suggesting that lymphocyte GSH is one of the molecular targets of chloramines. In conclusion, the results emphasize the relationships between PMN and lymphocytes, two major cell types in host defences. The former are a source of chloramines, and can downregulate lymphocyte proliferation through the target molecule, glutathione. Although several mechanisms resulting in decreased lymphocyte function and subsequent immunodepression are plausible, a chloramine-mediated pathway is an attractive hypothesis in various situations involving PMN-induced oxidative stress, that may have therapeutic implications. Due to the importance of lymphocytes in the immunologic response, this work has been focused on the consequences of chloramine scavenging by GSH in lymphocytes. However, we can speculate that this mechanism by which cell GSH is depleted could have different effects in other cell types.

### References

1. Klebanoff SJ. Oxygen metabolites from phagocytes. In: Gallin JI, Golstein IM, Snyderman R, eds. *Inflammation: Basic Principles and Clinical Correlates*. 2nd edn. New York: Raven Press Ltd, 1992.
2. Babior BM. Oxygen-dependent microbial killing by phagocytes. *N Engl J Med* 1978; **298**: 659–668.
3. Nathan CF. Neutrophil activation on biological surfaces. Massive secretion of hydrogen peroxide in response to products of macrophages and lymphocytes. *J Clin Invest* 1987; **80**: 1550–1560.
4. Winterbourn CC. Neutrophil oxidants: production and reactions. In Das DK, Essman WB, eds. *Oxygen Radicals: Systemic Events and Diseases Processes.* Basel: Karger, 1990; 31–70.
5. Weiss SJ, Lampert MB, Test ST. Long-lived oxidants generated by human neutrophils: characterization and bioactivity. *Science* 1983; **222**: 623–628.
6. Farber JL, Kyle ME, Coleman JB. Biology of disease. Mechanisms of cell injury by activated oxygen species. *Lab Invest* 1990; **62**: 670–679.
7. Swaim MW, Pizzo S. Methionine sulfoxide and the oxidative regulation of plasma proteinase inhibitors. *J Leukocyte Biol* 1988; **43**: 365–379.
8. Wasil M, Halliwell B, Hutchinson DCS, Baum H. The antioxidant action of human extracellular fluids. *Biochem J* 1987; **243**: 219–223.
9. El-Hag A, Lipsky PE, Bennett MRA, Clark RA. Immunomodulation by neutrophil myeloperoxidase and hydrogen peroxide: differential susceptibility of human lymphocyte functions. *J Immunol* 1986; **136**: 3420–3426.
10. Staite ND, Messner RP, Zoschke DC. Inhibition of human T lymphocyte E rosette formation by neutrophils and hydrogen peroxide. *J Immunol* 1987; **139**: 2424–2430.
11. Meister A, Anderson ME. Glutathione. *Ann Rev Biochem* 1983; **52**: 711–760.
12. Messina JP, Lawrence DA. Cell cycle progression of glutathione-depleted human peripheral blood mononuclear cells is inhibited at S phase. *J Immunol* 1989; **143**: 1974–1981.
13. Burchill BR, Oliver JM, Pearson CB, Leinbach ED, Berlin RD. Microtubule dynamics and glutathione metabolism in phagocytosing human polymorphonuclear leukocytes. *J Cell Biol* 1978; **76**: 439–447.
14. Gougerot-Pocidalo MA, Fay M, Roche Y, Lacombe P, Marquetty C. Immune oxidative injury induced in mice exposed to normobaric $O_2$: effects of thiol compounds on the splenic cell sulfhydryl content and Con-A proliferative response. *J Immunol* 1985; **135**: 2045–2051.
15. Hamilos DL, Zelarney Z, Mascali JJ. Lymphocyte proliferation in glutathione-depleted lymphocyte: direct relationship between glutathione availability and the proliferative response. *Immunopharmacology* 1989; **18**: 223–235.
16. Nguyen AT, Golub R, Feuillet-Fieux MN, Descamps-Latscha B. Modulation of human granulocyte and monocyte chemiluminescence responses: evidence for distinct free radical generating systems. *J Clin Lab Med* 1983; **12**: 47–55.
17. Witko V, Nguyen AT, Descamps-Latscha B. Microtitre plate assay for phagocyte-derived taurine-chloramine. *J Clin Lab Anal* 1992; **6**: 47–53.
18. Anderson ME. Determination of glutathione and glutathione disulfite in biological samples. *Methods Enzymol* 1985; **113**: 548–555.
19. Weiss SJ. Tissue destruction by neutrophils. *N Engl J Med* 1989; **320**: 365–376.
20. Halliwell B, Gutteridge JMC. In: *Free Radicals in Biology and Medicine*, 2nd edn. Oxford: Clarendon Press, 1989; 86–187.

21. Nathan CF, Arrick BA, Murray HW, DeSantis NM, Cohn ZA. Tumor cell anti-oxidant defenses. Inhibition of the glutathione redox cycle enhances macrophage-mediated cytosis. *J Exp Med* 1980; **153:** 766–782.
22. Harlan JM, Levine JD, Callahan KS, Schwartz BR, Harker LA. Glutathione redox cycle protects cultured endothelial cells against lysis by extracellularly generated hydrogen peroxide. *J Clin Invest* 1984; **73:** 706–713.
23. Ishii Y, Partridge CA, Del Vecchio PJ, Malik AB. Tumor necrosis factor-$\alpha$-mediated decrease in glutathione increases the sensitivity of pulmonary vascular endothelial cells to $H_2O_2$. *J Clin Invest* 1992; **89:** 794–802.
24. Bilzer M, Lauterburg BH. Effects of hypochlorus acid and chloramines on vascular resistance, cell integrity, and biliary glutathione disulfide in the perfused rat liver: modulation by glutathione. *J Hepatol* 1991; **13:** 84–89.
25. Smyth MJ. Glutathione modulates activation-dependent proliferation of human peripheral blood lymphocyte populations without regulating their activated function. *J Immunol* 1991; **146:** 1921–1927.
26. Liang CM, Lee N, Cattell D, Liang SM. Glutathione regulates interleukin-2 activity on cytotoxic T-cells. *J Biol Chem* 1989; **264:** 13519–13523.
27. Liang SM, Lee N, Finbloom DS, Liang CM. Regulation by glutathione of interleukin-4 activity on cytotoxic T-cells. *Immunology* 1992; **75:** 435–440.

ACKNOWLEDGEMENTS. The authors thank Françoise Tresset for her excellent technical assistance, Mare Thevenin and Irène Ceballos-Picot for suggestions concerning GSH determination and Carl Nathan for helpful comments and suggestions in the discussion of our manuscript. This work was supported by a grant from the Association Français de Lutte contre la Mucoviscidose (AFLM).

**Received 16 March 1993; accepted 5 April 1993**
